# Standardizing Feeding Strategies in Moderately Preterm Infants

**DOI:** 10.21203/rs.3.rs-2520889/v1

**Published:** 2023-02-06

**Authors:** Ting Ting Fu, Maame Arhin, Ashley Schulz, Laura Ward, Stacie Chapman, Abigail Adamchak, Jae Kim

**Affiliations:** Cincinnati Children’s Hospital Medical Center; Cincinnati Children’s Hospital Medical Center; University of Cincinnati College of Medicine; Cincinnati Children’s Hospital Medical Center; University of Cincinnati Medical Center; University of Cincinnati Medical Center; University of Cincinnati

## Abstract

**Objective:**

To evaluate the impact of a standardized feeding protocol and donor breast milk (DBM) provision on clinical outcomes in moderate preterm infants (MPT, 29–33 6/7 weeks gestational age).

**Study Design:**

A protocol for MPT infants born > 1500 g was implemented clinically to standardize feeding advancements at 30 mL/kg/day. Infants < 33 weeks received DBM. We retrospectively identified 131 and 144 infants born before and after implementation. Clinical data including central venous line (CVL) placement, feeding tolerance, growth, and provision of maternal breast milk (MBM) were collected.

**Result:**

Number of CVLs, days to full enteral volume (FEV), and MBM provision was unchanged. There was a narrower range of days to FEV post-implementation. Growth metrics were similar between eras.

**Conclusion:**

Implementation of a feeding protocol for MPT infants is associated with more consistent time to FEV With monitoring and appropriate fortification, DBM use in this population is not associated with worse growth outcomes.

## Introduction

Strategies implemented in the neonatal intensive care unit (NICU) to reduce the incidence of necrotizing enterocolitis (NEC) have largely focused on the higher risk population of very low birth weight (VLBW) infants (birth weight less than 1500 grams).^1, 2^ Two such strategies are standardized feeding protocols and use of pasteurized donor breast milk (DBM) when maternal breast milk (MBM) is not available.^3, 4, 5, 6^ Although the incidence of NEC is lower at baseline in moderately preterm (MPT) infants − 2.4% in infants born at 29 through 33 weeks versus 7.6% in VLBW infants – there is the potential that standardized feeding and DBM may still provide some advantage to higher gestational age non-VLBW infants.^7, 8^

To date, there are no published data regarding standardizing feeding exclusively in the MPT population. Studies that examined the implementation of a standardized feeding regimen that included both infants born less than and greater than 1500 grams did not provide stratified results.^3, 9, 10, 11, 12, 13^ Without a standardized feeding protocol in place for these larger infants, medical providers may be inconsistent, with some still opting for slower feeding advancements, which could require central venous access to provide parenteral nutrition (PN) to optimize nutrient delivery until higher feeding volumes are attained. Presence of a central line increases the risk for a bloodstream infection despite vigilance during placement and maintenance procedures. However, no difference in incidence of NEC or death has been demonstrated with faster versus slower feeding advancements.^14, 15^ Therefore, implementation of a standardized feeding protocol in theory could safely reduce the necessity for a central venous line (CVL) and improve morbidity.

The impact of the availability of DBM on clinical outcomes has also primarily been investigated in VLBWs, though human milk is the preferred nutrition for all preterm infants.^16, 17^ The presence of DBM in NICUs in the United States has increased in the last decade, but there is significant variation eligibility and duration of use.^17, 18, 19, 20, 21^ Extending the provision of DBM to larger preterm infants who would otherwise receive preterm formula, either as supplementation to MBM or primary diet, offers them the unique benefits of human milk that currently cannot be mimicked otherwise, though it remains unclear whether there is a measurable clinical difference. However, one of the primary concerns regarding the use of DBM is its nutritional composition and impact on growth outcomes, though appropriate growth can be achieved with fortification and milk processing strategies.^5, 22, 23, 24^ The evidence is mixed whether the provision of MBM is unchanged or increased by DBM availability, but some studies report a higher rate of direct breastfeeding at discharge.^25, 26, 27, 28^ As these studies focused on the VLBW population, it is unknown if these findings can be generalized to MPT infants.

Thus, we aim to review the utilization of a standardized feeding protocol and DBM in MPT infants to characterize and compare clinical outcomes and growth trajectories before and after implementation.

## Methods

This retrospective study was conducted at a level III NICU in Cincinnati, Ohio, and was approved by the Cincinnati Children’s Hospital Institutional Review Board with a waiver of authorization. In January 2019, to standardize practice and reduce variation, the NICU implemented a feeding protocol for preterm infants born less than 34 weeks completed gestation and with birth weight greater than 1500 grams. At the same time, the eligibility for DBM was increased from VLBW to include all infants born less than 33 weeks completed gestation. This feeding protocol was developed as a corollary to an established standardized feeding protocol for VLBW infants that requires placement of a central venous line for total parenteral nutrition.^29^ For this MPT protocol, enteral feedings are initiated within 24 hours of life at 20 mL/kg/day while awaiting the provision of MBM, then advanced by 15 mL/kg/day every 12 hours. At 110 mL/kg/day, intravenous fluids are stopped, human milk is fortified to 24 kcal/oz, and feedings are advanced by 10 mL/kg/day every 12 hours until goal. Due to the pace of the protocol, central venous access is not required, and peripheral PN was prescribed when applicable. See Appendix 1 for more details of the feeding protocol.

Infants born weighing more than 1500 grams and between 29 0/7 and 33 6/7 weeks gestational age (following the Neonatal Research Network definition) were identified from the 18 month period prior to and after implementation. Infants who died or transferred in the first week of life were excluded. For all eligible infants, demographic and clinical information was collected from the medical chart, including placement of a CVL (primary outcome), length of stay, days receiving PN or intravenous fluids, positive blood culture, and diagnosis of NEC (any Bell’s stage). Nutritional data of interest included first feeding substrate, highest caloric density for fortification, days to achieve full enteral volume (FEV), whether each infant received any MBM or was receiving MBM at discharge, and whether each infant ever directly breastfed or was directly breastfeeding at discharge. DBM and MBM intake was calculated by dividing the total volume of DBM or MBM intake by the total enteral intake during the entire NICU hospitalization. Anthropometric measurements obtained at birth, 28 days (if still hospitalized), and discharge were recorded and converted to Fenton z-scores.^30^ Body mass index (BMI) was calculated and converted to Olsen z-score.^31^ Growth velocities were calculated from birth to discharge and from birth to 28 days. Weight velocity was calculated using the average 2-point method.

Statistical analysis was performed using SAS software version 9.4 (SAS Institute Inc., Cary, NC, USA). Pre-and post-implementation clinical outcomes and growth metrics were compared by Chi-squared, Mann Whitney U, and t-tests. Results were considered statistically significant for p values < 0.05. Since DBM eligibility was limited to less than 33 weeks, infants born between 33 and 34 weeks gestational age were excluded for nutrition and growth analyses.

## Results

131 and 144 eligible MPT infants were identified in the pre- and post-implementation eras respectively. Out of these, 82/131 and 99/144 were born prior to 33 weeks completed gestation and were offered DBM as supplementation to MBM. Table 1 shows the demographic and overall clinical outcomes, which were similar for the two groups. Compliance with initiation of the MPT feeding protocol in the post-implementation era was 90%, with 9.7% of infants still receiving a CVL specifically for PN due to slower feeding advancements. Of the five cases of late onset sepsis in the post-implementation cohort, none were associated with the presence of a CVL. Incidence of NEC was similar between eras (2.3% pre vs. 3.5% post, p = 0.56). Of the infants diagnosed with NEC, one patient from each era received slower feeding advancements per the VLBW protocol.

**Figure 1:**
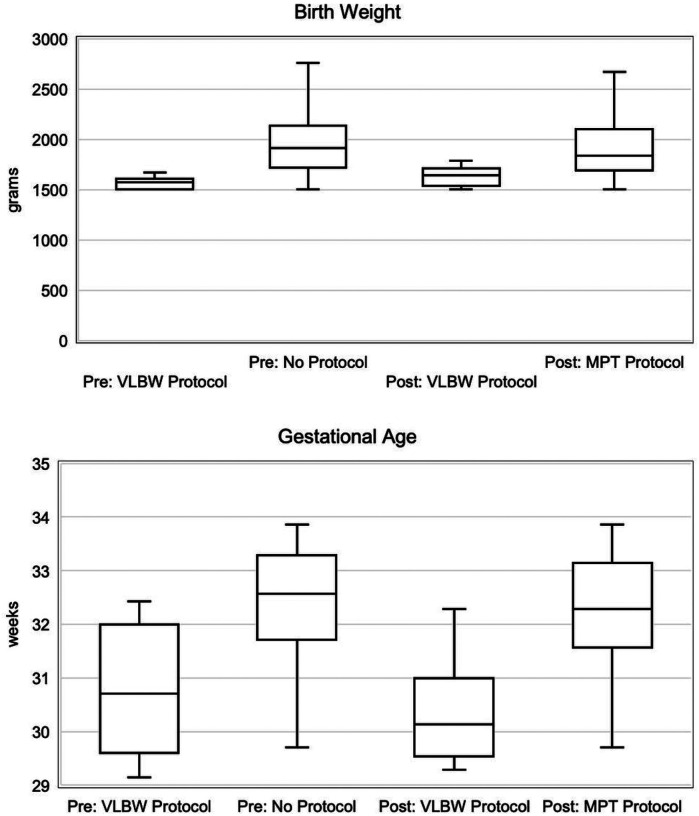
Boxplot distributions of birth weight and gestational age, grouped by feeding protocols, before and after implementation. Pre (VLBW Protocol) n=8, Pre (No Protocol) n=123, Post (VLBW Protocol) n=14, Post (MPT Protocol) n=130.

Examining the eight infants pre-implementation and the fourteen infants post-implementation who received a CVL to support a slower feeding advancement, there was no statistically significant difference between the eras in either median birth weight (1573.0 g (IQR 1514.9–1603.0) pre vs. 1644.0 g (IQR 1553.1–1701.0) post, p = 0.12) or gestational age (30.7 weeks (IQR 30.0–31.7) pre vs. 30.1 weeks (IQR 29.6–30.9) post, p = 0.43) (Fig. 1). When compared to the 253 infants who received a faster feeding advancement, those 22 infants collectively were smaller in median birth weight (1593.0 g (IQR 1518.1–1670.1) pre vs. 1868.0 g (IQR 1708.1–2120.0) post, p < 0.0001) and gestational age (30.4 weeks (IQR 29.6–31.0) pre vs. 32.4 weeks (IQR 31.7–33.3) post, p < 0.0001). However, in both eras, the range in birth weight and gestational age of infants placed on the slower VLBW protocol overlapped with those who were advanced faster (Fig. 1).

**Figure 2:**
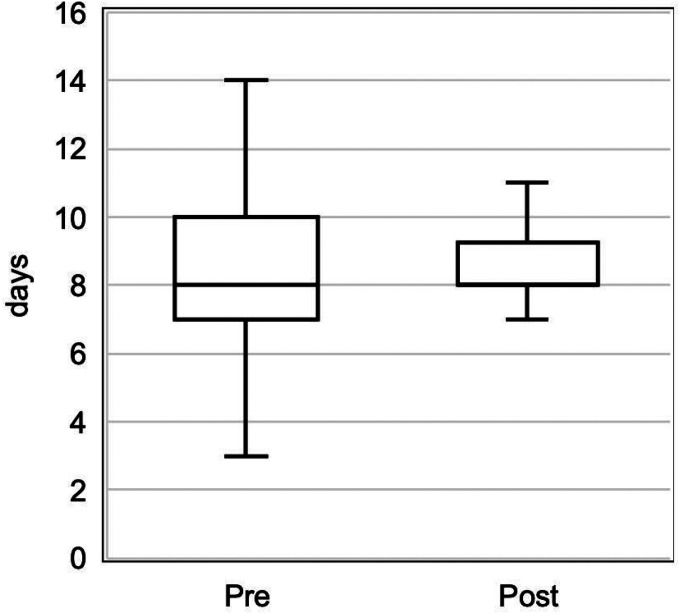
Boxplots of days to full enteral volume feedings, before and after implementation. Pre n=82, Post n=99.

Table 2 presents the nutrition outcomes between the two eras. With the extended eligibility of DBM post-implementation, there was a higher incidence of human milk at first feeding (67.1 % pre vs. 89.9% post, p < 0.001) and percentage of DBM intake (1.4% pre vs. 17.5% post, p < 0.01). Days to FEV was unchanged, but there was a narrower range post-implementation (7–11 days pre vs. 8–10 days post) (Fig. 2). Rates of initiation or sustainment of either MBM expression or direct breastfeeding was not impacted.

Table 3 shows the growth velocities and changes in z-scores over time, which were not statistically different before and after implementation. For infants whose length of stay was greater than 28 days, length velocity was modestly decreased in the first 28 days (1.05 cm/week pre vs. 0.86 cm/week post, p = 0.08) with the availability of DBM. For this subset of patients, this difference in linear growth was not observed out to discharge (1.02 ± 0.29 cm/week pre vs. 0.92 ± 0.30 cm/week post, p = 0.19).

## Discussion

In this study, we demonstrated 90% compliance with implementation of a standardized feeding protocol for MPT infants but did not find a decrease in the frequency of CVL placement. This was likely due to sustained perception from some members of the clinical team that smaller and younger preterm infants remain at increased risk for NEC and may benefit from slower feeding advancements. However, this concern and subsequent non-compliance was inconsistent, as there was overlap in the gestational age and birth weight ranges of infants in each protocol group.

Jasani and Patole previously performed a systematic review that examined the implementation of a standardized feeding regimen for preterm infants. While the fifteen studies were all observational, their meta-analysis of 18,160 infants found that a standardized feeding regimen was associated with a pooled relative risk for NEC of 0.22.^3^ Five of the fifteen studies included preterm infants up to 2000 or 2500 grams, though they were older (1978 to 2006) and did not separate VLBW from larger infants to provide stratified results.^3, 9, 10, 11, 12, 13^ Kamitsuka et al. did group the cases of NEC by gestational age (greater than or equal to 32 weeks vs. less than 30 weeks) but did not provide subgroup sizes.^10^ To our knowledge, our study is the first to evaluate the use of a standard feeding protocol exclusively in preterm infants who don’t fall under the higher risk VLBW categorization. We identified eight total cases of NEC in the MPT population in this three-year span (2.9%), consistent with the Neonatal Research Network.^7^ Although we observed that the incidence of NEC was unchanged after implementation, we suspect this was due to our low baseline rate. Importantly, standardized feeding advancements at 30 mL/kg/day was not associated with an increase in the incidence of NEC. This is consistent with a Cochrane systematic review and the SIFT trial, which both found no difference in incidence of NEC or death with faster (30–40 mL/kg/day) versus slower feeding advancements in VLBW and very preterm infants,^14, 15^ who potentially have decreased gut maturity and integrity compared to MPT infants. These data support exploring faster rates of standardized feeding advancement (>30 mL/kg/day) for moderate preterm infants to reduce dependency on vascular access.

Furthermore, the limited number of studies on feeding protocol implementation that included larger infants all reported some degree of formula use. Brown and Sweet’s study in 1978 described a regimen that used strictly water and formula with no mention of human milk.^9^ Kamitsuka et al. and Street et al. both reported a mixture of formula and MBM, with no indication of DBM.^10, 11^ These studies predated the growing availability of DBM as supplementation to MBM.^21^ This is an important consideration as human milk is associated with both improved feeding tolerance and a decreased incidence of NEC.^32, 33^ Feeding tolerance is likely impacted by certain human milk components that enhance maturation of the gastrointestinal tract and improve gut motility, and though pasteurization may alter bioactivity levels, some growth factors and signaling molecules remain intact and unaffected by processing.^34, 35, 36^ Additionally, whey proteins, which are easier to digest than casein, are found in higher proportion in human milk compared to formula.^37^ Proteins specific to human milk may also have innately different digestibility and absorption in infants compared to bovine proteins.^38^ Many of these benefits of unpasteurized human milk have been extrapolated to DBM, and its use has expanded beyond the VLBW population without much published data on outcomes for these larger and higher gestational age infants.^16, 20^ In our case, the expanded eligibility criteria for DBM was well-accepted by the unit, and overall we encountered minimal barriers and no adverse events. In utilizing days to FEV as proxy for feeding tolerance, we showed no difference between cohort eras but did find a narrower range and interquartile range after implementation.

In contrast, Kamitsuka et al. reported an increase in time to reach FEV, while Street et al. saw no difference in days to reach half FEV or 80 mL/kg/day, their unit’s volume at which CVLs were removed; again, both these studies differed in their population from ours and included smaller infants.^10, 11^

Suboptimal growth is consistently a concern associated with the use of DBM, though with appropriate fortification strategies, adequate growth has been demonstrated in VLBW infants.^5, 22, 23, 24^ Here we present novel evidence that anthropometric velocities can be maintained with the introduction of DBM for MPT infants. However, noting that 41–44% of our cohorts received fortification beyond 24 kcal/oz, we recognize that our unit’s neonatal dietitians are particularly sensitive to the detection of growth faltering and liberally increase the fortification density in response to suboptimal growth. This may be an important aspect of generalizing our results to other NICUs. While there also appeared to be a trend towards a small difference in linear growth for infants who were admitted for at least 28 days, the clinical significance of a difference of 0.2 cm/week is unclear. Furthermore, this difference was not observed out to discharge, and at the time, a recumbent measuring board for obtaining weekly length was not routinely used, a limitation to our study. The availability of DBM was also not associated with a change in rates of MBM provision at discharge or direct breastfeeding at discharge, adding to the conflicting findings reported in the literature.^25, 26, 27, 28^

Another limitation to our study is that the MPT feeding protocol was implemented at the same time as the expanded DBM eligibility criteria, thus making it difficult to tease out their individual effect on feeding tolerance as an outcome. In the SIFT trial, there was a small degree of statistical interaction between the feeding substrate (human milk, formula, or a mixture) and the rate of feeding advancement, and the authors speculated that different diets may have unique risk-benefit profiles with regards to feeding strategies.^14^ We advocate that both a standardized feeding approach and the availability of DBM are valuable to patient outcomes and family satisfaction, and we have demonstrated they are safe to implement collectively for MPT infants. Other limitations include the retrospective nature of our study, the convenience sampling for patient selection, and the focus on short-term outcomes. One additional clinical nutritional change occurred during the middle of the pre-implementation era: the upper cutoff for initiating dextrose infusions containing amino acids was increased from a birth weight of 1750 grams to 2500 grams.

In conclusion, implementation of a feeding protocol with standardized volume advancements and DBM use is associated with a more consistent time to FEV without impacting incidence of NEC in MPT infants. With monitoring and appropriate fortification, DBM use in this population does not appear to impact growth negatively. Further prospective studies evaluating feeding practices for MPT infants are warranted.

## Figures and Tables

**Table 1:** Infant characteristics and clinical outcomes, before and after implementation

	Pre (n = 131)	Post (n = 144)	p-value
Birth weight (grams)	1888.1 (1687.9–2127.9)	1798.9 (1669.1–2034.5)	0.19
Gestational age at birth (weeks)	32.4 (31.7–33.3)	32.1 (31.4–33.1)	0.14
Gestational age at discharge (weeks)	35.7 (35.0–36.7)	35.7 (34.9–36.9)	0.97
Sex (male) (%)	72 (55%)	90 (62.5%)	0.20
Length of stay (days)	23 (16–32)	24 (18–33.5)	0.28
Central venous line placement (any during admission) (%)	17 (13%)	26 (18.1%)	0.25
Central venous line placement (for slower feeding advancement) (%)	8 (6.1%)	14 (9.7%)	0.27
Necrotizing enterocolitis (any Bell’s stage) (%)	3 (2.3%)	5 (3.5%)	0.56
Late onset sepsis (%)	1 (0.8%)	5 (3.5%)	0.12
Days receiving parenteral nutrition or intravenous fluids	4.7 (3.3–5.9)	4.1 (3.2–5.3)	0.12

Median (IQR)

**Table 2:** Nutrition outcomes for infants born < 33 weeks, before and after implementation

	Pre (n = 82)	Post (n = 99)	p-value
First feeding human milk (%)	55 (67.1%)	89 (89.9%)	<0.001
Fortification >24 kcal/oz (%)	34 (41.5%)	44 (44.4%)	0.69
Days to full enteral feeding volume	8 (7–11)	8 (8–10)	0.70
% donor milk intake	1.4 (0–39.7)	17.5 (1.3–46.5)	<0.01
% maternal milk intake	73.1 (15.6–97.0)	58.3 (23.4–95.7)	0.88
Mother initiated pumping (%)	72 (87.8%)	80 (80.8%)	0.20
Receiving maternal milk at discharge (%)	45 (54.9%)	46 (47.9%)^1^	0.35
Direct breastfed ever (%)	47 (57.3%)	51 (51.5%)	0.44
Direct breastfed within 72 hours of discharge (%)	19 (23.2%)	15 (15.6%)^1^	0.20

Median (IQR)

1 3 infants transferred prior to discharge, n = 96

**Table 3:** Growth outcomes for infants born < 33 weeks, before and after implementation

	Birth to Discharge			Birth to 28 Days		
	Pre (n = 82)	Post (n = 99)	p-value	Pre (n = 36)	Post (n = 45)	p-value
Weight velocity (g/day)	21.07 ± 8.68	21.37 ± 8.71	0.82	26.57 ± 4.98	26.30 ± 6.46	0.84
Length velocity (cm/week)	0.81 ± 0.52	0.80 ± 0.57	0.91	1.05 ± 0.44	0.86 ± 0.37	0.08
HC velocity (cm/week)	0.72 ± 0.38	0.64 ± 0.38	0.20	0.75 ± 0.23	0.71 ± 0.31	0.63
Change in weight z-score	−0.65 ± 0.37	−0.67 ± 0.45	0.79	−0.60 ± 0.43	−0.56 ± 0.42	0.72
Change in length z-score	−0.61 ± 0.65	−0.68 ± 0.69	0.50	−0.49 ± 0.75	−0.77 ± 0.62	0.11
Change in HC z-score	−0.28 ± 0.72	−0.35 ± 0.82	0.52	−0.33 ± 0.69	−0.38 ± 0.94	0.81
Change in BMI z-score	−0.42 ± 0.76	−0.36 ± 0.99	0.69	−0.45 ± 1.09	−0.04 ± 0.89	0.11

Mean ± SD

## Data Availability

The data presented in this study are available on request from the corresponding author.
